# COVID-19 and Gastrointestinal Disease: Current Insights and Future Management

**DOI:** 10.3390/jcm12072727

**Published:** 2023-04-06

**Authors:** Jonathan Kopel, Hemant Goyal

**Affiliations:** 1 Department of Internal Medicine, Texas Tech University Health Sciences Center, Lubbock, TX 79430, USA; jonathan.kopel@ttuhsc.edu; 2 Center for Interventional Gastroenterology at UT (iGUT), Department of Endoluminal Surgery & Interventional Gastroenterology, The University of Texas Health Sciences Center, 6431 Fannin Street, Houston, TX 77030, USA

## 1. Introduction

The first case of coronavirus disease 2019 (COVID-19) was reported in Wuhan, Hubei Province, China, in December 2019, marking a pivotal moment in human history. The seventh coronavirus known to infect people is SARS-CoV-2 which causes coronavirus disease 2019 (COVID-19). Multimorbid patients greater than 50 years of age are more likely to die from COVID-19. The most common symptoms of COVID-19 include high fever, cough, and myalgia. Atypical gastrointestinal (GI) symptoms were recognized in COVID-19 patients a little later and appeared to be associated with severe disease. Patients can continue to shed the virus even after the resolution of pulmonary symptoms. Therefore, patients with GI symptoms are often evaluated for COVID-19. SARS-CoV-2 has recently been linked to fecal–oral transmission in the USA and China, proving that it can multiply in both the digestive and respiratory tracts. Moreover, the epidemiology, clinical characteristics, diagnostic procedures, treatments, and prevention of the gastrointestinal manifestations of COVID-19 remain to be elucidated.

Despite therapeutic advances, particularly vaccines, the COVID-19 pandemic remains a global health issue. Among the various organ systems affected, the effects of SARS-CoV-2 on the GI system are widely recognized. Beyond the classic symptoms of nausea, vomiting, and diarrhea, SARS-CoV-2 can cause damage to the pancreas and liver, resulting in acute pancreatitis and deranged liver tests. However, the epidemiology, pathogenesis, and treatment of the GI effects of SARS-CoV-2 remain an active area of investigation. GI endoscopists are at increased risk of exposure to and/or transmission of SARS-CoV-2. As such, it was not uncommon for endoscopists to defer elective endoscopic procedures during the beginning of the COVID-19 pandemic. This Special Issue explores the challenges that SARS-CoV-2 poses to gastroenterologists and endoscopists in their daily practice.

### 1.1. Effects of SARS-CoV-2 on the Gastrointestinal System in Hospitalized COVID-19 Patients

Although liver tests remain a primary method for assessing liver function, deviations in laboratory tests for liver injury secondary to SARS-CoV-2 infection are observed frequently among gastroenterologists. Tokarczyk et al. performed a retrospective study to analyze several liver tests (aspartate aminotransferase, alanine aminotransferase, alkaline phosphatase, gamma-glutamyltransferase, total bilirubin, and albumin) of admitted COVID-19 patients in the context of other comorbidities and risk factors to assess their predictive value for the need for intensive care unit admission, mechanical ventilation necessity, and mortality [1]. The authors investigated whether these markers alone or combined with other measures, such as the neutrophil-to-lymphocyte ratio, could help assess the risk of in-hospital mortality. Their analysis suggests that albumin may help predict the hospital course of COVID-19 patients, particularly those with hypoalbuminemia. Tokarczyk et al.’s study suggests that using liver tests may aid in assessing the overall course of hospitalized COVID-19 patients.

Zelenika et al. performed a fascinating study in which they examined whether transient elastography (TE) to measure patients’ liver stiffness measurement (LSM), controlled attenuation parameter (CAP), and FibroScan-AST (FAST) score could be used with other non-invasive surrogates of liver steatosis to predict the clinical severity and 30-day composite outcome of hospitalized COVID-19 patients [2]. The authors conducted a retrospective study of 217 COVID-19 patients and found that neither LSM nor CAP correlated with COVID-19 severity or outcomes. However, the FAST score was shown to be an independent risk factor for 30-day mortality or the need for mechanical ventilation. A study by Pausawasdi et al. examined the clinical outcomes of non-COVID-19 patients hospitalized for upper gastrointestinal bleeding (UGIB) during the pandemic [3] and found that compared to pre-pandemic times, hospitalized non-COVID UGIB patients were older, had more underlying malignancies, and had lower platelet and albumin levels during the pandemic. Esophagogastroduodenoscopy (EGD) was also performed less frequently in non-COVID-19 UGIB patients despite prolonged hospitalization and increased 30-day mortality and rebleeding rates [3]. Using a retrospectively collected cohort, Kaliszewski et al. showed that abdominal pain alone or with dyspnea did not increase the risk of a worse clinical course or mortality among hospitalized COVID-19 patients [4]. Together, these studies indicate that combining liver tests with gastrointestinal imaging could provide an effective method for assessing and predicting the morbidity and mortality risk for hospitalized COVID-19 patients.

### 1.2. Gastrointestinal Complications among Hospitalized COVID-19 Patients

As the COVID-19 pandemic progressed, long-term complications related to the GI system continued to emerge and be recognized. From a large multicenter registry of 53,682 inflammatory bowel disease (IBD) patients, Zabana et al. identified 482 COVID-19 patients between March and July 2020 to study their disease course and clinical evolution over 12 months. They found that age >60 years (OR 7.1) and >2 comorbidities (OR 3.9) were associated with mortality which, was 3.7% in the cohort. Interestingly, the use of steroids in the previous 3 months before COVID-19 diagnosis was the only predictor of physical sequelae such as myalgia, arthralgia, and asthenia at 3 months [5]. To evaluate the impact of COVID-19 on the psychiatric health of IBD patients, Ryu et al. performed an electronic survey in South Korea and found significant anxiety and depression related to COVID-19. Unvaccinated status, female gender, and the presence of psychiatric illness were associated with greater risks of anxiety and depression. Immunomodulator use was associated with a higher risk of anxiety [6]. Clerbaux et al. performed a literature review and found that SARS-CoV-2 could induce intestinal inflammation by binding to ACE2 and infecting intestinal bacteria [7,8]. In addition, Serban et al. and Łykowska-Szuber et al. examined the literature to show the relationships between acute mesenteric ischemia and deranged liver tests among hospitalized COVID-19 patients [9,10]. The above publications illustrate the complications associated with SARS-CoV-2 infection and provide further insights into COVID-19 and various common GI diseases.

## 2. Conclusions and Future Challenges Faced by Gastroenterologists

As Tontini et al. reiterated, the COVID-19 pandemic has ushered in a series of challenges for gastroenterologists in terms of the diagnosis, treatment, and prevention of long-term complications among hospitalized COVID-19 patients [11]. Due to the COVID-19 pandemic, there has been an increase in hospitalization, interdisciplinary complexity, length of hospital stay, and mortality among COVID-19 patients. These lessons reveal a great need for the development of adequate safety, management, and long-term surveillance procedures for gastrointestinal complications within this patient population.
